# Highly restrictive and directional penetration of the blood cerebral spinal fluid barrier by JCPyV

**DOI:** 10.1371/journal.ppat.1012335

**Published:** 2024-07-22

**Authors:** Bethany A. O’Hara, Avraham S. Lukacher, Kaitlin Garabian, Jacob Kaiserman, Evan MacLure, Hiroshi Ishikawa, Horst Schroten, Sheila A. Haley, Walter J. Atwood

**Affiliations:** 1 Department of Cell Biology, Biochemistry, and Molecular Biology, The Warren Alpert Medical School, Brown University, Providence, Rhode Island, United States of America; 2 University of Tsukuba, Tsukuba City, Japan; 3 Department of Pediatrics, Medical Faculty Mannheim, Mannheim, Germany; University of Wisconsin-Madison, UNITED STATES OF AMERICA

## Abstract

The human polyomavirus JCPyV is an opportunistic pathogen that infects greater than 60% of the world’s population. The virus establishes a persistent and asymptomatic infection in the urogenital system but can cause a fatal demyelinating disease in immunosuppressed or immunomodulated patients following invasion of the CNS. The mechanisms responsible for JCPyV invasion into CNS tissues are not known but direct invasion from the blood to the cerebral spinal fluid via the choroid plexus has been hypothesized. To study the potential of the choroid plexus as a site of neuroinvasion, we used an adult human choroid plexus epithelial cell line to model the blood-cerebrospinal fluid (B-CSF) barrier in a transwell system. We found that these cells formed a highly restrictive barrier to virus penetration either as free virus or as virus associated with extracellular vesicles (EV^JC+^). The restriction was not absolute and small amounts of virus or EV^JC+^ penetrated and were able to establish foci of infection in primary astrocytes. Disruption of the barrier with capsaicin did not increase virus or EV^JC+^ penetration leading us to hypothesize that virus and EV^JC+^ were highly cell-associated and crossed the barrier by an active process. An inhibitor of macropinocytosis increased virus penetration from the basolateral (blood side) to the apical side (CSF side). In contrast, inhibitors of clathrin and raft dependent transcytosis reduced virus transport from the basolateral to the apical side of the barrier. None of the drugs inhibited apical to basolateral transport suggesting directionality. Pretreatment with cyclosporin A, an inhibitor of P-gp, MRP2 and BCRP multidrug resistance transporters, restored viral penetration in cells treated with raft and clathrin dependent transcytosis inhibitors. Because choroid plexus epithelial cells are known to be susceptible to JCPyV infection both in vitro and in vivo we also examined the release of infectious virus from the barrier. We found that virus was preferentially released from the cells into the apical (CSF) chamber. These data show clearly that there are two mechanisms of penetration, direct transcytosis which is capable of seeding the CSF with small amounts of virus, and infection followed by directional release of infectious virions into the CSF compartment.

## Author summary

In patients with compromised immune systems due to underlying immunosuppressive diseases or due to treatment with immunomodulatory drugs, JC virus (JCPyV) invades the brain and causes a rapidly progressing and often fatal demyelinating disease known as progressive multifocal leukoencephalopathy (PML). The brain is protected from invading pathogens by two major barriers, the blood-brain barrier (BBB), and the blood-cerebrospinal fluid barrier (BCSFB). The BCSFB is composed of choroid plexus epithelial cells that are directly exposed to the blood. The epithelial cells in the choroid plexus are held together by tight junctions that limit and regulate the movement of molecules and cells from the blood to the cerebral spinal fluid. In this paper we used an established adult choroid plexus epithelial cell line to model the blood-cerebrospinal fluid barrier and found that it was highly restrictive of virus penetration. The restriction was not absolute and small amounts of virus penetrated and established infection in underlying human glial cells. We also show a second mechanism of virus invasion involving direct infection of choroid plexus epithelial cells by JCPyV and directional release of infectious virions across the barrier. Both mechanisms likely contribute to the neuroinvasiveness of JCPyV.

## Introduction

JCPyV is the causative agent of a rapidly progressing and often fatal demyelinating disease known as progressive multifocal leukoencephalopathy (PML) [1]. Originally associated with HIV/AIDS and classified as an AIDS defining illness, PML has emerged in more recent years as a complication in multiple sclerosis patients being treated with highly effective immunomodulatory drugs [2]. The incidence of PML in HIV patients is currently 1.3 per 100,000 [3]. The overall incidence of PML in the MS patient population is estimated at 3 per 100,000 [3]. Unlike the early days of the AIDS pandemic when PML was uniformly fatal, the majority (75%) of patients diagnosed with PML survive, although they are left with significant disabilities [3]. There are no specific antiviral therapies against JCPyV although immune checkpoint inhibitor and virus-specific T cell therapies are showing promise [4–6]. The majority of these approaches rely on the restoration of immune function which can result in immune reconstitution inflammatory syndrome (IRIS), which can worsen PML symptoms [7]. PML-IRIS occurs in both HIV/AIDS and MS patients, in particular in patients treated for longer periods with natalizumab (Tysabri) [8]. PML has also been associated with other autoimmune conditions that are treated with immunomodulatory drugs, including Crohn’s disease, lupus, psoriasis and some cancers. A growing list of immunomodulatory drugs now carry PML warnings and eight of these have been given FDA black box labeling in the United States [9]. Utilization of these immune-modulating therapies is widespread, resulting in an increase in drug-associated PML over the past several years [10,11]. Recent genome-wide association studies identified four genetic variants that increase the risk of developing PML by ten-fold in patients taking immunomodulatory drugs representing the only significant biomarker for determining risk other than serostatus and antibody indices [12]. Understanding how the virus penetrates blood-CSF and blood-brain barriers could lead to new strategies to prevent initiation of disease in at-risk populations.

The choroid plexus is a highly vascularized organ in the central nervous system (CNS) responsible for producing cerebrospinal fluid (CSF) and for controlling immune cell trafficking into the CNS [13]. The endothelial cells lining the vessels in the choroid plexus are fenestrated allowing direct contact of peripheral blood with choroid plexus epithelium (CPE), polarized cells which form a tight barrier between the blood and the CSF [14]. The blood-CSF barrier is also a site where pathogens including several neurotropic viruses can invade the CNS [15–19]. In earlier studies we demonstrated that primary choroid plexus epithelial cells are fully susceptible to infection with the human polyomavirus JCPyV [20]. In addition, JCPyV infection of the choroid plexus has been shown in patients with PML [21,22]. We also recently demonstrated that infection of primary choroid plexus epithelial cells results in significant increases in secretion of inflammatory chemokines and subsequent downregulation of proteins involved in maintaining tight junctions [23].

Because primary choroid plexus epithelial cells are not optimal for these studies we used an established adult choroid plexus cell line to mechanistically study direct virus penetration of the barrier either as free virus or as virus associated with extracellular vesicles [24]. We found that small amounts of virus either as free virus or as extracellular vesicle associated virus was capable of directly penetrating the barrier and that this was not by passive paracellular transport but rather by active transcytosis. Penetration by virus was also found to be directional, as inhibitors of transcytosis preferentially inhibited entry from the basolateral to the apical chamber which represents movement from the blood to the CSF. Transcytosis inhibitors blocked viral penetration but not that of EV associated virus, suggesting that the mechanism used by EVs to cross the barrier is fundamentally different from the mechanisms used by virions. Because choroid plexus epithelial cells are known to be susceptible to JCPyV infection both in vitro and in vivo we also modeled virus penetration from an infected cell barrier. We found that the infected cells released virus preferentially into the apical (CSF) chamber while the barrier remained intact. Both mechanisms likely contribute to the neuroinvasiveness of JCPyV.

## Results

### Establishment of the barrier

**Fig 1:**
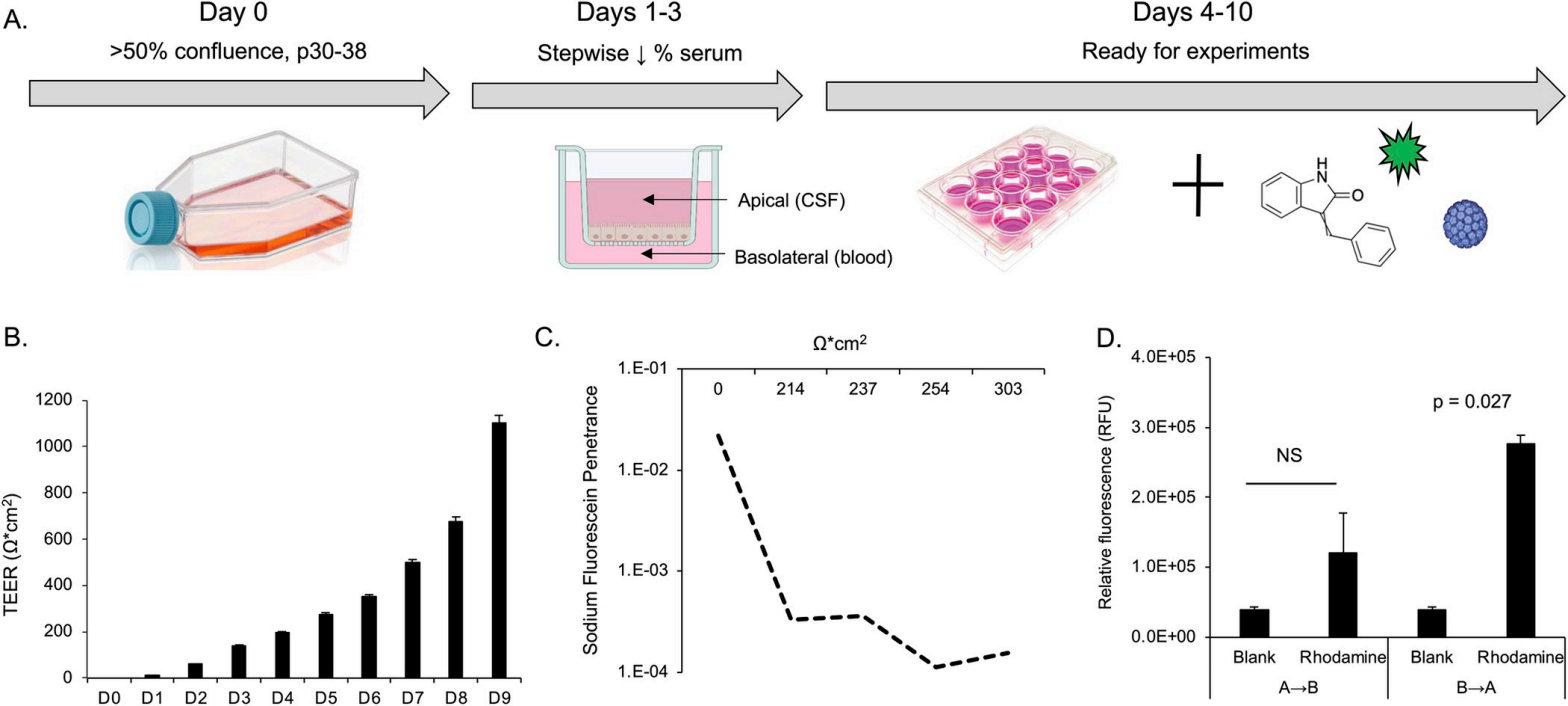
Establishment and characterization of a choroid plexus barrier system. A) Barrier development protocol. Cells are maintained at high density (>50% confluence) and used for experiments between passages 30–38. Cells are seeded to transwell inserts and follow a stepwise serum reduction protocol with daily monitoring until ready for experiments. Transwell cultures persist with sufficiently high TEER values for up to ten days. B) Representation of TEER values over time. TEER increases steadily over time up to day 9. C) Penetration of sodium fluorescein (SF), a small molecule tracer dye. SF was used to measure the rate of penetrance (cm/sec) from the apical to basal chamber over one hour. As TEER increases, penetrance decreases, indicating that a restrictive barrier has formed. D) Bidirectional transport of rhodamine 123. Relative fluorescence (RFU) was measured in the apical and basal chambers after 3 hours. Rhodamine is preferentially transported in the basolateral to apical direction (B to A) rather than apical to basolateral (A to B) indicating the barrier is polarized. Error bars in panels B and D represent the standard deviation between three independent experiments, in triplicate. Images in Fig 1A, were created with BioRender.com under license to Brown University.

The human choroid plexus papilloma cell line (HIBCPP) was used to mimic a choroid plexus epithelial cell barrier. Cells were grown on transwell inserts and the barrier was established by following a stepwise reduction in serum (Fig 1A). An EVOM-2 voltohmmeter was used to measure transepithelial electrical resistance (TEER) across the membrane as an indicator of barrier integrity (Fig 1B). TEER values increased steadily over time indicating the formation of an intact barrier (Fig 1B). Above 300 ohms*cm^2^, sodium fluorescein (SF) penetration was restricted, proportional to increasing TEER (Fig 1C). The cells forming the barrier were also found to be polarized as they preferentially transported labeled rhodamine-123 from the basal to the apical chamber (Fig 1D).

### The HIBCPP barrier is highly restrictive of JCPyV penetration either as purified virus or when associated with EV

**Fig 2:**
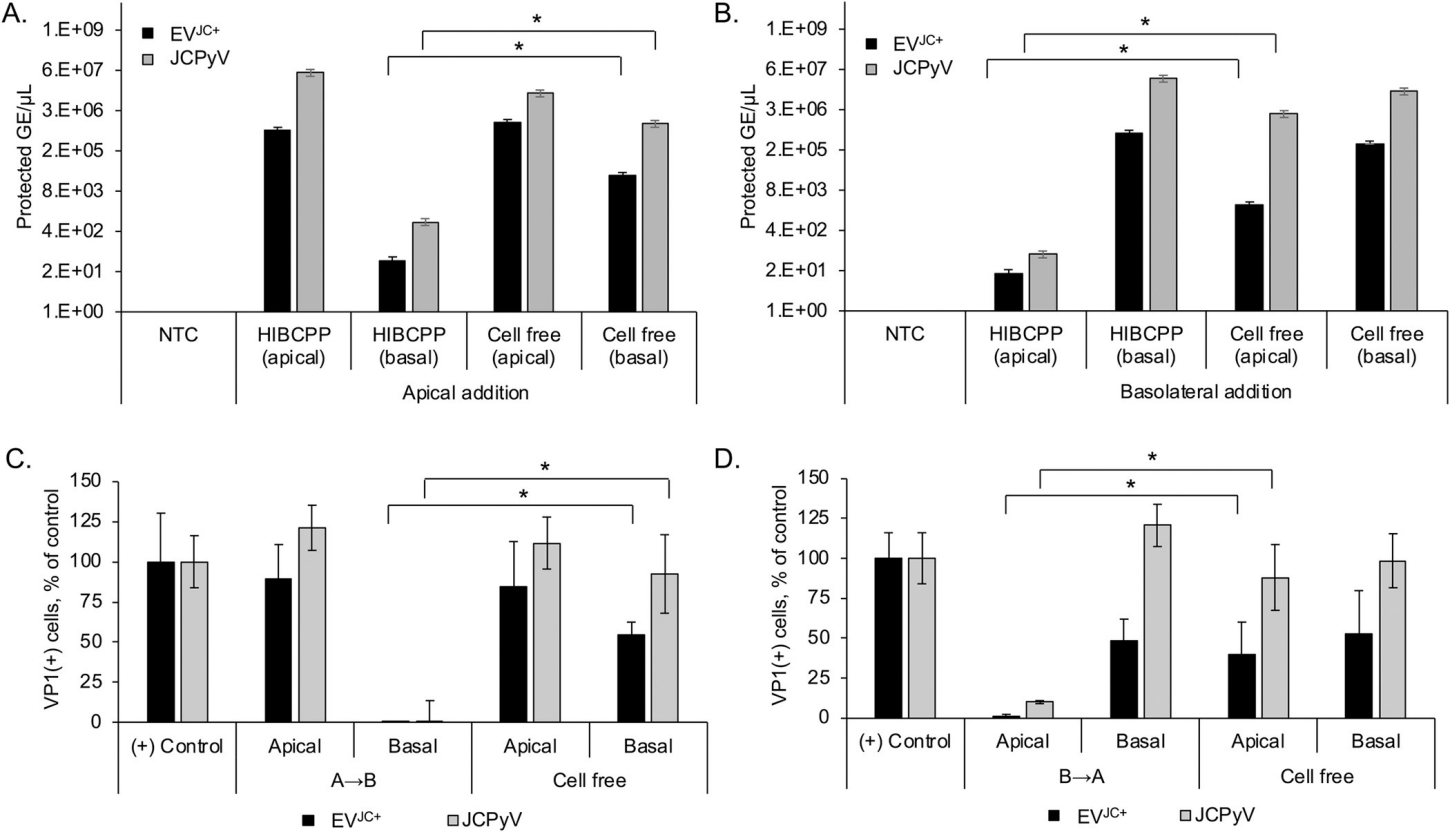
HIBCPP barrier cultures significantly restrict JCPyV and EV^JC+^. A) Barriers were set up on transwell inserts as described. 3^10^/ml genomes of JCPyV or EV^JC+^ were added to the apical cell surface of HIBCPP barrier cultures and viral genomes were quantified by qPCR from both apical and basal chamber supernatants after 48h. B) JCPyV or EV^JC+^ were added to the basolateral cell surface of HIBCPP barrier cultures and viral genomes were quantified from both apical and basal chamber supernatants after 48h. C-D) Apical and basal supernatants from panels A and B were used to infect SVG-A cells and scored for VP1 at 3 days post infection. The majority of JCPyV or EV^JC+^ is restricted to the chamber in which its added. * = p < 0.05. NTC = no template control. (B to A), basolateral to apical direction. (A to B) apical to basolateral direction. Infection with purified JCPyV or EV^JC+^ was used as the positive (+) control, to compare the spread of infection. Error bars represent the standard deviation between three independent experiments, in triplicate.

**Fig 3:**
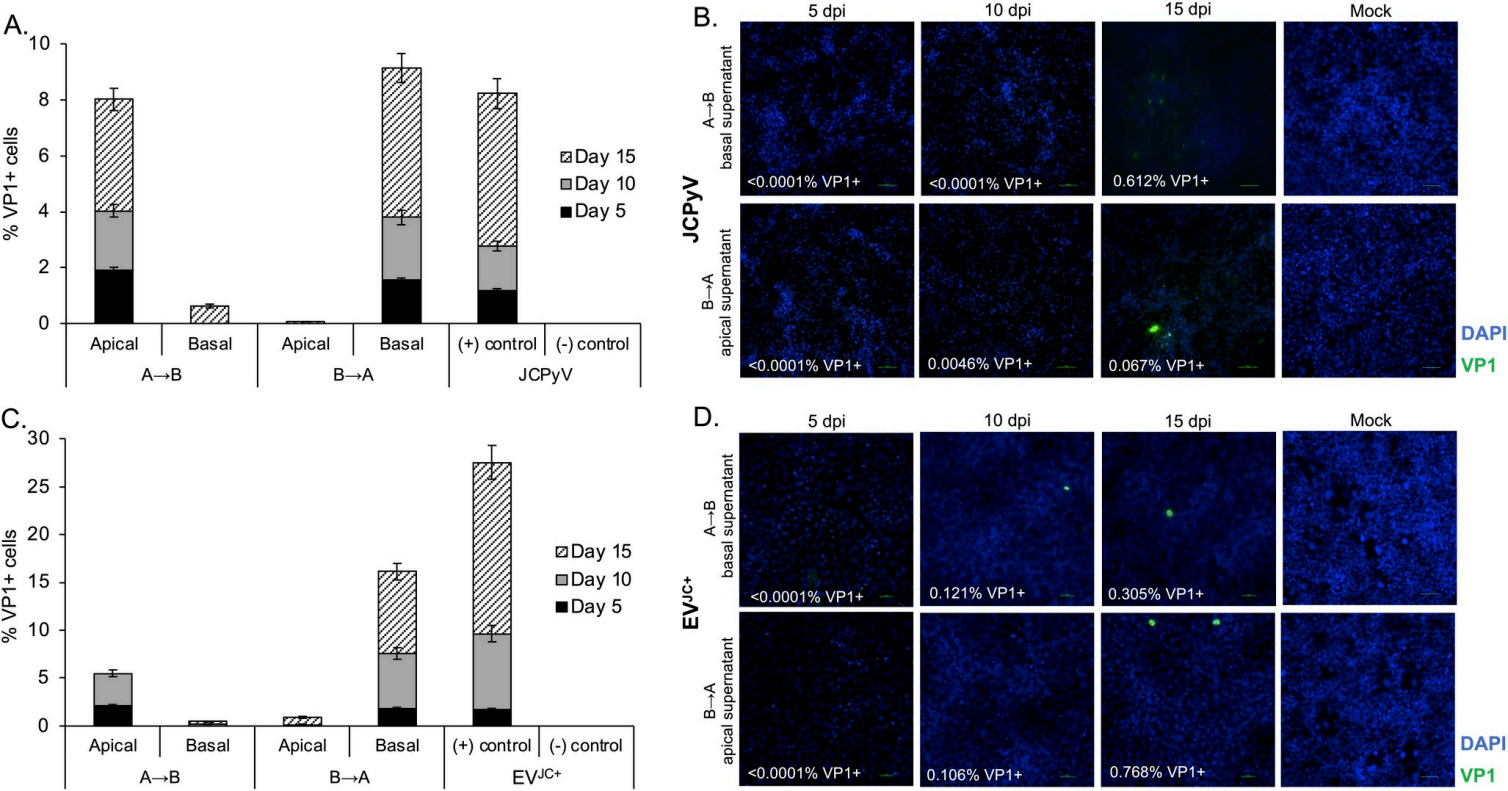
Barrier-infiltrating JCPyV and EV^JC+^ establish infected foci in primary human astrocytes following multiple rounds of replication. Supernatants from the barrier infiltration experiment shown in Fig 3 were used to reinfect primary human astrocytes (NHA) and allowed to replicate for 15 days. Apical and basal supernatants were collected after 48h incubation with virus or EV^JC+^ and used to infect NHA. The infection was monitored for viral spread over time. A) VP1 expression in NHA following barrier infiltration by JCPyV at days 5, 10 and 15 post infection. VP1+ cells increase over time. B) Clusters of infected cells are apparent by day 15. C) VP1 expression in NHA following barrier infiltration by EV^JC+^ at days 5, 10 and 15 post infection. VP1+ cells increase over time. D) Clusters of infected cells are apparent by day 15. DAPI (total cell count) is shown in blue and VP1 (infected cell count) is shown in green. Mock = SVG-A cells incubated with supernatant from naïve HIBCPP cells from day 7, and stained for VP1 at 15dpi. Virus is detectable at day 15 by both indirect immunofluorescence and qPCR, and is able to spread over time. (B to A), basolateral to apical direction. (A to B) apical to basolateral direction. Infection with purified JCPyV was used as the positive (+) control, to compare spread. Mock infection with viral diluent alone (DMEM/F12 media) was used as the negative (-) control. Error bars represent the standard deviation between three independent experiments, in triplicate.

HIBCPP barriers were established and then exposed to purified JCPyV or to EV^JC+^ either in the apical (panels 2A and 2C) or the basolateral (panels 2B and 2D) chambers for 48 hours. Penetration of the barrier by virus or EV^JC+^ was measured by assaying each chamber for protected viral genomes using quantitative PCR. When cells were absent from the transwell, both virus and EV^JC+^ equilibrated on both sides of the membrane indicating that the membrane by itself did not restrict passage (Fig 2, Cell Free controls in each panel). A no template control was also used to control for PCR contamination (Fig 2, NTC in panels A and B). The majority of virus or EV^JC+^ failed to penetrate and remained in the chamber in which it was added (Fig 2A and 2B). The relative amount of virus that does penetrate the barrier to the underlying chamber is detectable by quantitative PCR (Fig 2A and 2B). Supernatants from each chamber were then used to infect SVG-A cells and a minimal level of infection was detected at 3 days post-infection from the small amount of virus or EV^JC+^ that had penetrated from either side (Fig 2C and 2D). When allowed to spread over multiple rounds of infection, this low level of barrier-infiltrating JCPyV and EV^JC+^ was adequate to cause foci of JCPyV positive cells, which increased with time (Fig 3).

### Penetration of the barrier is an active process rather than by passive paracellular transport

**Fig 4:**
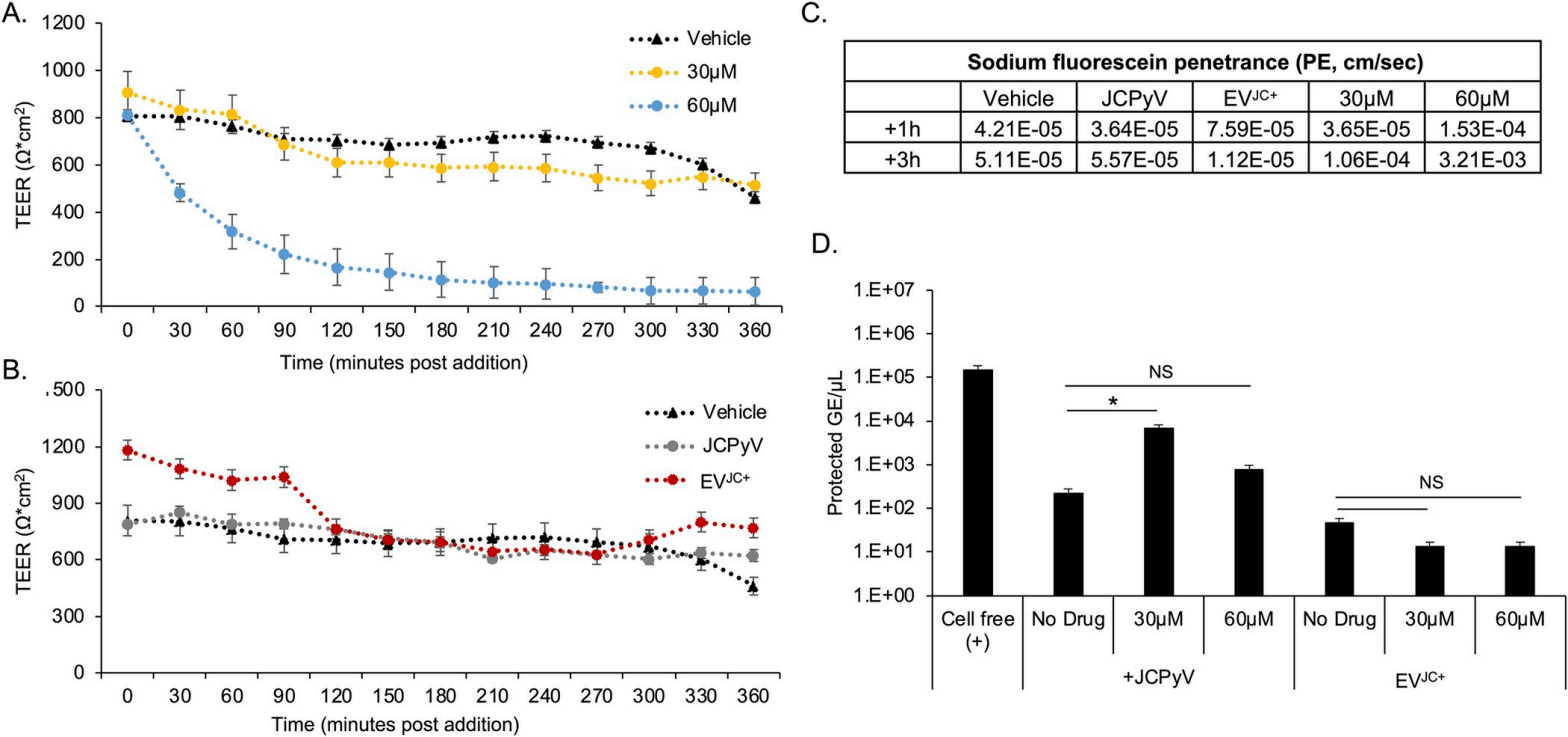
Intercellular disruption of HIBCPP barriers does not increase penetration of JCPyV or EV^JC+^. A) Following initial TEER readings, vehicle control (DMSO) or 30μM and 60μM capsaicin were added to barrier cultures. TEER readings were taken every 30 minutes, starting at time zero, for 6 hours. Capsaicin disrupted intercellular barrier integrity in a dose dependent manner. B) Following initial TEER readings, JCPyV, vehicle control, or EV^JC+^ were added to barrier cultures. TEER readings were taken every 30 minutes, starting at time zero, for 6 hours. Addition of JCPyV, vehicle or EV^JC+^ did not disrupt barrier integrity. C) Sodium fluorescein penetrance was calculated following 1 hour and 3 hour exposure to capsaicin, JCPyV, EV^JC+^ or vehicle. As expected, PE increases following barrier incubation with capsaicin. D) Quantification of viral penetration by qPCR after capsaicin treatment. NS = not significant, * = p < 0.05. Error bars represent the standard deviation between three independent experiments, in triplicate.

**Fig 5:**
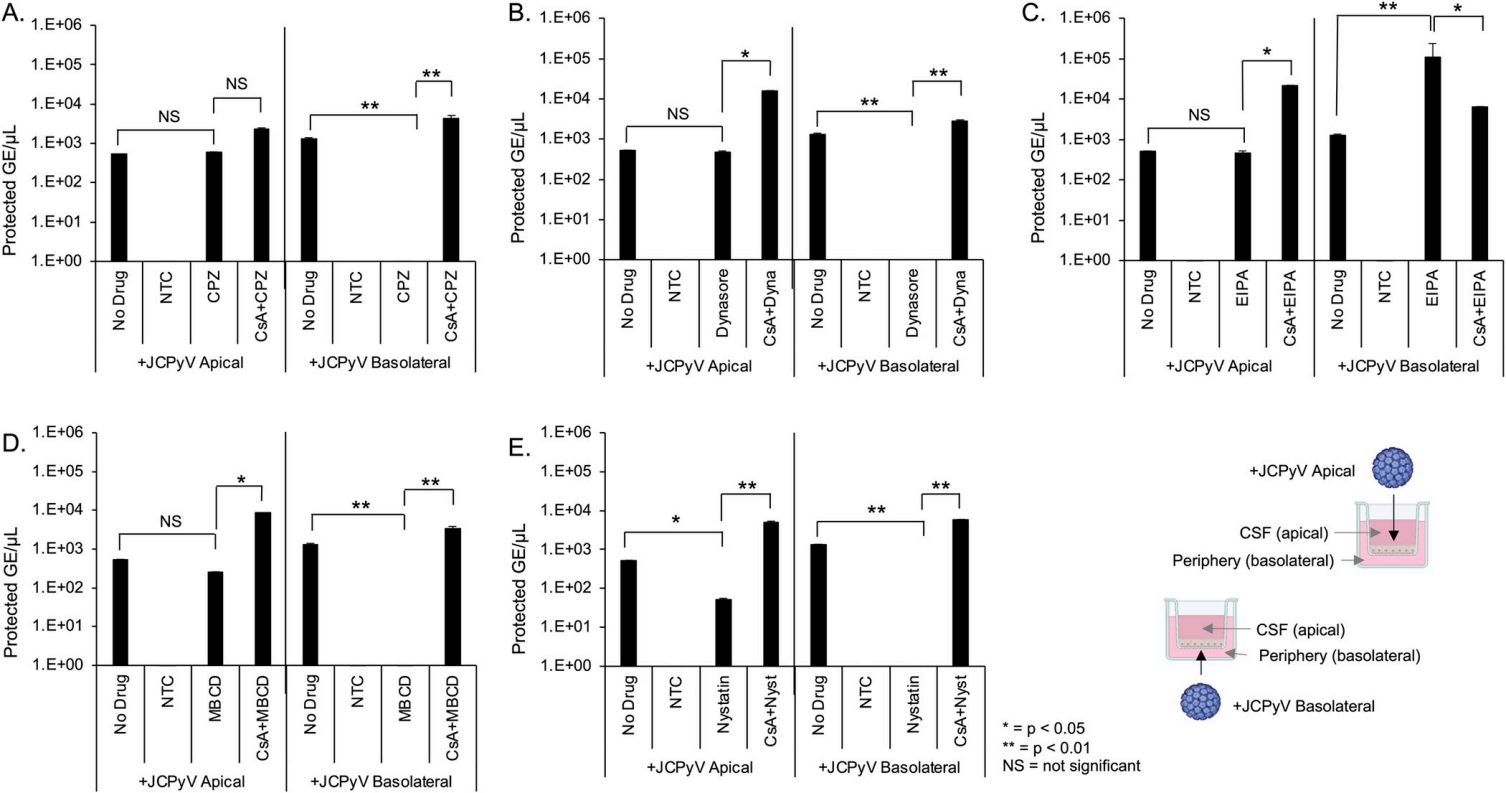
The effect of transcytosis inhibitors on barrier penetration by JCPyV. HIBCPP cells were cultured on transwell inserts as described and treated with transcytosis inhibitors alone, or cyclosporin A for one hour followed by inhibitors. 3^10^/ml genome copies of purified JCPyV was added to either the apical or basal chamber for an additional 24h. A) qPCR quantification of JCPyV barrier penetration following treatment with 100μM chlorpromazine. B) qPCR quantification of JCPyV barrier penetration following treatment with 100μM dynasore. C) qPCR quantification of JCPyV barrier penetration following treatment with 100μM EIPA. D) qPCR quantification of JCPyV barrier penetration following treatment with 5mM methyl-beta-cyclodextran (MßCD). E) qPCR quantification of JCPyV barrier penetration following treatment with 5μM nystatin. Cyclosporin A (CsA) restored virus penetration from the basolateral to apical chambers to equivalent with the untreated control (no drug, panels A-E). NS = not significant, * = p < 0.05, ** = p < 0.01. NTC = no template control. Error bars represent the standard deviation between three independent experiments, in triplicate. Images in Fig 5E were created with BioRender.com under license to Brown University.

Pathogens can breach the BCSF barrier when the barrier is compromised by inflammatory processes, by direct infection and destruction of the epithelial cells, or by transcytosis with little to no disruption of the barrier. To distinguish between these possibilities, we artificially disrupted the barrier using capsaicin. Capsaicin reversibly disrupts tight junctions in epithelial cells by dephosphorylation of cofilin and reorganization of F-actin [25, 26]. Capsaicin treatment of HIBCPP barriers resulted in a dose dependent reduction in TEER relative to vehicle control (Fig 4A). Sodium fluorescein penetration increased in the capsaicin treated transwells in a dose dependent manner (Fig 4C). We also asked whether exposure to virus or virus contained in EV would disrupt barrier integrity. Treatment of the barrier with JCPyV and EV^JC+^ had no effect on TEER or sodium fluorescein penetration (Fig 4B and 4C). We next measured whether JCPyV or EV^JC+^ would penetrate the barrier more efficiently following disruption by capsaicin. JCPyV and EV^JC+^ penetration of the barrier was detectable by qPCR but was not increased by capsaicin despite the increased permeability of the barrier (Fig 5D). This suggests viral migration across the barrier is an active process rather than via paracellular transport and is consistent with the highly cell associated nature of polyomaviruses. Capsaicin does not have significant anti-JCPyV activity and was used at non-toxic doses (S2 Fig).

### Inhibitors of transcytosis preferentially affect virus penetration across the barrier

**Fig 6:**
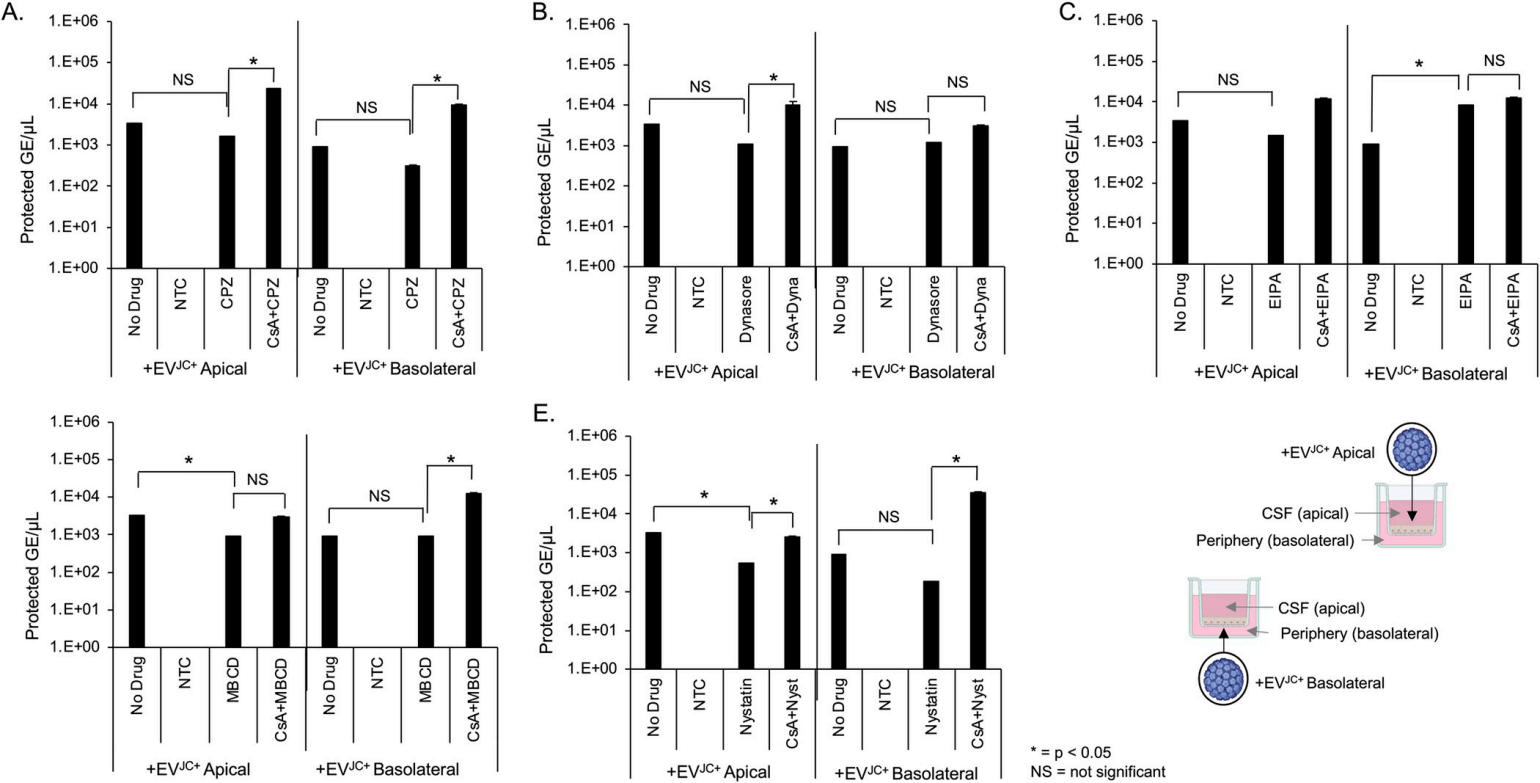
The effect of transcytosis inhibitors on barrier penetration by EV^JC+^. HIBCPP cells were cultured on transwell inserts as described and treated with transcytosis inhibitors alone, or cyclosporin A for one hour followed by inhibitors. 3^10^/ml genome copies of EV^JC+^ was added to either the apical or basal chamber for an additional 24h. A) qPCR quantification of EV^JC+^ barrier penetration following treatment with 100μMchlorpromazine. B) qPCR quantification of EV^JC+^ barrier penetration following treatment with 100μM dynasore. C) qPCR quantification of EV^JC+^ barrier penetration following treatment with 100μM EIPA. D) qPCR quantification of EV^JC+^ barrier penetration following treatment with 5mM methyl-beta-cyclodextran (MßCD). E) qPCR quantification of EV^JC+^ barrier penetration following treatment with 5μM nystatin. Inhibitors did not block basal to apical penetration of EV^JC+.^ NS = not significant, * = p < 0.05. NTC = no template control. Error bars represent the standard deviation between three independent experiments, in triplicate. Images in Fig 6E were created with BioRender.com under license to Brown University.

We next asked whether virus or EV^JC+^ penetrated the barrier by active transcytosis. We used drugs to target clathrin dependent (chlorpromazine and dynasore), lipid raft/caveolae dependent (MßCD and nystatin), and macropinocytosis (EIPA) dependent transport. Transwell cultures were pretreated with inhibitors for 2 hours, followed by addition of JCPyV or EV^JC+^ in the continued presence of drug. After 24 hours, supernatant from the opposing chamber were analyzed by qPCR for protected genome content. Chlorpromazine, nystatin, MßCD and dynasore blocked basolateral to apical transport of virus but had no effect on apical to basolateral trafficking (Fig 5A, 5B, 5D and 5E). EIPA enhanced virus trafficking from basolateral to apical and had no effect on apical to basolateral transport (Fig 5C). Pretreatment of barrier cultures with cyclosporin A was able to restore viral penetration to the level of untreated samples (Fig 5A–5E). The tested inhibitors had very little impact on the transcytosis of EV^JC+^ suggesting an alternate mechanism of barrier penetration by EV^JC+^ than by free virus (Fig 6). All compounds were used at non-toxic doses and the relative pathway targeting ability of each drug was determined by their ability to inhibit uptake of control fluorescent molecules (S3 Fig). TEER and sodium fluorescein penetrance were not negatively impacted by incubation with these inhibitors (Figs S4 and S5).

### Dissemination of virus from infected choroid plexus barriers

**Fig 7:**
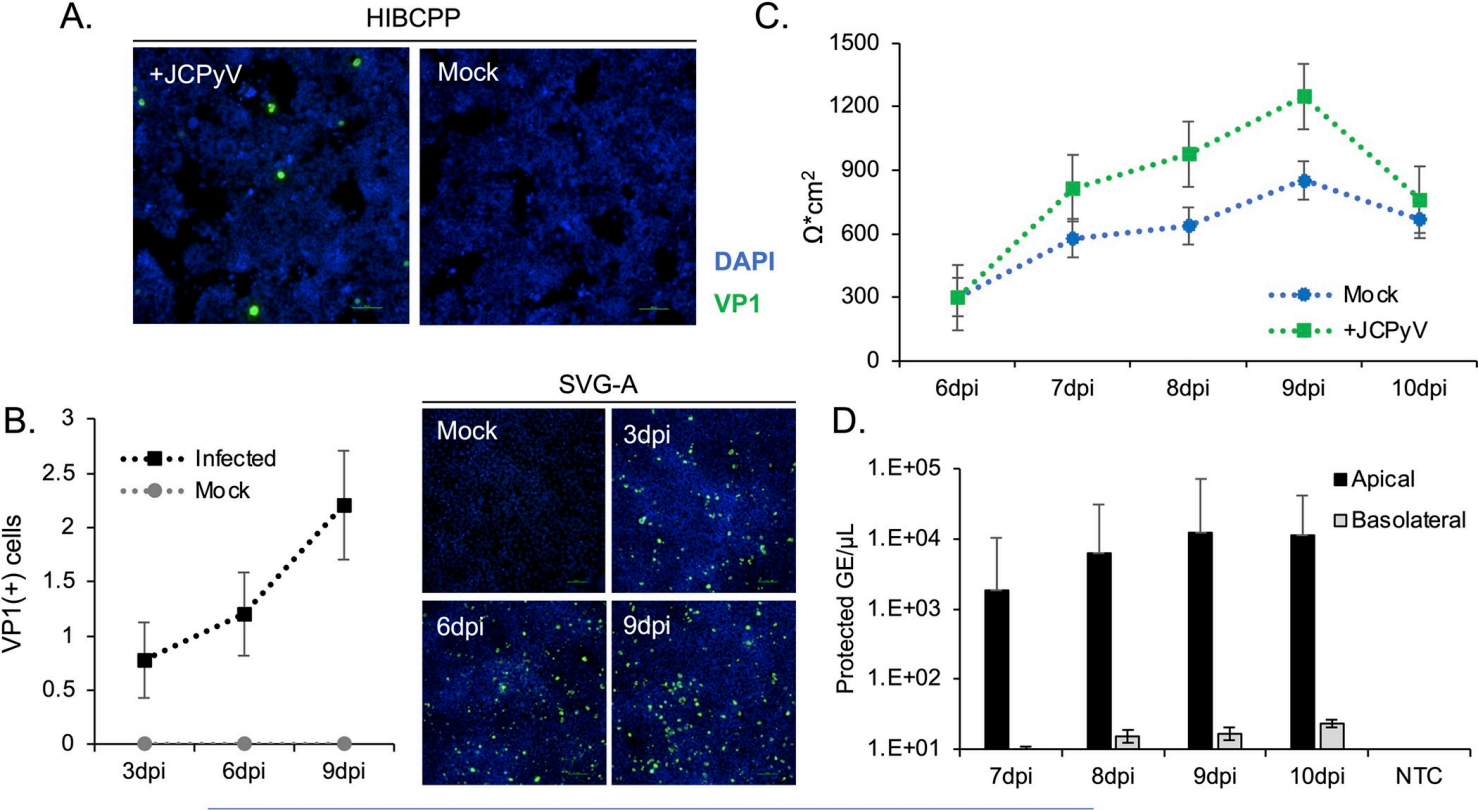
Release of infectious virions from JCPyV+ HIBCPP barriers. A) HIBCPP monolayers were infected with JCPyV for 2 hours. Three days post infection, infected and mock infected cells were plated to transwell culture dishes and 96 well plates. HIBCPP cells were stained for VP1 at 7dpi to verify the presence of a productive infection. VP1+ cells are shown in green; DAPI was used to calculate a total cell count and is shown in blue. Scale bar = 100μm. B) Media collected from day 7 was used to infect SVG-A cultures in a growth assay over 15 days. HIBCPP cells release infectious virions capable of establishing an infection and spreading in target cells. VP1+ cells are shown in green; DAPI was used to generate a total cell count and is shown in blue. Scale bar = 100μm. C) TEER was monitored daily and is shown as Ω*cm^2^. Values were calculated by subtracting the TEER reading of cell free control wells from that of the sample wells, and multiplying by the transwell surface area. D) A sample of media from the apical and basal chambers was collected daily and analyzed for protected viral genome content by qPCR. Virus is preferentially released into the apical chamber. Error bars represent the standard deviation between three independent experiments, in triplicate. Mock = mock infected cells, negative control; DPI = days post infection; NTC = no template control.

HIBCPP cells were exposed to JCPyV and the infected cells plated to transwell inserts and to 96-well plates at three days post-infection. The susceptibility of the cells to infection with JCPyV was confirmed by staining the infected cells growing in the 96-well plate with antibodies to VP1 (Fig 7A). Supernatants from the infected HIBCPP cells were also used to infect SVG-A glial cells and after several rounds of infection, significant viral spread is apparent (Fig 7B). The infected HIBCPP cells growing in the transwell dishes were monitored for barrier integrity daily for 10 days post infection (7 days after seeding transwells) using TEER. The barrier remained intact, despite being infected with JCPyV (Fig 7C). Supernatants were then collected from the apical and basal chambers of the transwell dished and analyzed by qPCR for protected viral genome content. Virus was preferentially released into the apical (CSF) chamber for several days following infection (Fig 7D).

## Discussion

The choroid plexus is recognized as a staging ground for the invasion of brain parenchyma by several viral pathogens including Echovirus 30, SARS CoV2, and HSV-1 [13,19,27,28]. The mechanisms of JCPyV neuroinvasion are not known but virus infection in choroid plexus epithelial cells both in vivo and in vitro has been documented [20,22,29]. These cells are also known to express the major receptors for JCPyV [30]. The gold-standard for diagnosis of JC virus induced PML is a positive PCR derived from the CSF using an ultra-sensitive multiplex assay [31,32]. Detection of very low amounts of JCPyV DNA in CSF are used as a diagnostic value for PML (as low as 25 copies/mL), highlighting that even small amounts of infiltrating virus are clinically relevant at the time of diagnosis [33–35]. Monitoring of viral loads and anti-JCPyV antibody levels are also routinely used as methods to determine extended interval dosing schedules in patients taking natalizumab, and assess overall risk [36]. However, the correlation between PML and viral load is not exact and as such, standard monitoring falls short in predicting disease onset. It is also not clear whether the virus in CSF derives from infected glia in brain parenchyma and drains to the CSF or whether it represents virus invading from the periphery; likely both occur during the progression of the disease. During periods of viremia, the potential for infection of choroid plexus cells on the basolateral surface may increase, eventually leading to a productive infection at this interface. This “tipping point” of infection and release of virions directly into the CSF, as shown in Fig 7, may help explain why JC viremia monitoring alone is not adequate for PML risk assessment.

In this report we focused our attention at defining viral neuroinvasion both through and originating from the blood-CSF barrier. JCPyV and many other viruses are released from infected cells both as “free” viral particles and EV associated virus [37–39]. A recent study showed JC virus like particles were able to transcytose a blood brain barrier model in vitro, as well as in a mouse model [40]. Similarly, we show evidence of viral transcytosis by both EV associated and non-EV associated virus that results in accumulation of clinically significant concentrations of virus in the CSF chamber. We utilized an established blood-CSF barrier model based on the growth of HIBCPP cells in a transwell culture system. As in healthy individuals, HIBCPP cells formed a restrictive barrier as measured by transepithelial electrical resistance and by their ability to restrict movement of a tracer dye, sodium fluorescein. The cells were also polarized as measured by directional transport of labeled rhodamine. Using this system, we asked whether purified virus or EV^JC+^ could penetrate the barrier. Following 24 hours exposure to JCPyV or EV^JC+^, the integrity of the barrier remained intact as evidenced by maintaining high TEER values, resistance to passage of sodium fluorescein, and restriction of the majority of both purified virus and EV^JC+^. The amount of barrier-infiltrating JCPyV and EV^JC+^ was sufficient to establish infectious foci in glial cells. Neither virus nor EV penetrated passively, as disruption of the barrier with capsaicin did not increase penetration. This is consistent with the highly cell-associated nature of polyomaviruses.

EV transcytosis can occur via multiple potential pathways [41]. To further define a mechanism for virus or EV transport across the barrier we treated cells with established inhibitors of clathrin dependent transcytosis (chlorpromazine and dynasore), raft-mediated endocytosis (nystatin and MßCD), and macropinocytosis (EIPA). Inhibitors and dosing were chosen based on previously demonstrated ability to specifically block JCPyV internalization and/or EV^JC+^ internalization [42–44]. All but EIPA limited transcytosis of purified virus from the basolateral chamber (periphery) to the apical chamber (CSF). EIPA, a macropinocytosis inhibitor, accelerated movement of virus from the basolateral chamber to the apical chamber, but not the reverse. Conversely, movement of virus from the apical to basolateral chamber was not impacted by any of the compounds tested. Chlorpromazine as well as several other drugs have been shown to interact with the ABC superfamily of transporters, both as inhibitors and substrates [44]. When activated, these transport proteins act to preferentially move substances out of the brain and back into the periphery [45–47]. This protective response is well established as a leading cause of drug resistance and chemotherapeutic failure [48,49]. By pretreating with cyclosporin A to interfere with the action of ABC transporters, we show a reversal in basolateral➔apical restriction of virus. This suggests a role for multidrug transporter proteins not only in restriction of antiviral drugs but also in the movement of JCPyV.

None of these inhibitors had an effect on EV mediated transcytosis suggesting a fundamentally different mechanism than that used by virus. EV uptake by recipient cells has been shown to occur via multiple pathways, including clathrin and caveolin -mediated endocytosis, phagocytosis, macropinocytosis and lipid raft-mediated endocytosis [29]. Given the ability of EV to exploit multiple routes of entry, it is not surprising that single drug treatment was unable to prevent EV^JC+^ invasion across the barrier. Recent work has shown that cells displaying multidrug resistance phenotype release more extracellular vesicles than their drug sensitive counterparts [50]; increased EV shedding following drug treatment could also play a role in EV^JC+^ barrier infiltration. Virus carrying EV are shown to be not only capable of delivering viruses to target cells, but also to enhance delivery in their ability to carry multiple viral particles in a single EV [51,52]. Previous work has shown that JCPyV is released by infected CPE cells and that this released JCPyV and EV^JC+^ are readily able to infect both receptor null and receptor bearing target cells in vitro, including human choroid plexus cells [29,53]. Pseudovirions with PML-associated mutations, lacking the ability to bind sialic acid (the necessary receptor for viral entry) are able to use EV to deliver their cargo to target cells, but cannot spread as purified virus [29]. In addition, EV associated virus is able to escape neutralization with anti-JCPyV antibodies. Given that major target cells in vivo lack the required receptors for JCPyV infection, and that EV are able to readily penetrate the blood-CSF, the potential for EV based neuroinvasion remains relevant.

Although we have shown how highly restrictive the B-CSF barrier model is, the choroid plexus itself can be infected with JCPyV in vivo [22]. We therefore established infection in the HIBCPP cells and then plated them to transwell dishes. Establishing the infection first was required because of the slow nature of JCPyV growth kinetics and the amount of time we can maintain the HIBCPP barrier in the transwells. We found that the infected HIBCPP cells were capable of forming a tight barrier and over the course of several days released significant amounts of infectious virus into the apical chamber (CSF side).

In summary, we show that free virus and EV-associated virus are able to penetrate an intact blood-CSF barrier model with low efficiency. The movement of purified virions across the barrier was blocked by inhibitors of clathrin and lipid-raft dependent transcytosis and movement was increased by an inhibitor of macropinocytosis. These drugs only effected basolateral to apical trafficking that represents movement from the blood to the CSF. None of the compounds impacted the transcytosis of EV^JC+^ suggesting an alternate mechanism of barrier penetration by EV^JC+^ than by free virus. We also show that infected choroid plexus epithelial cells preferentially release significant amounts of infectious virus from the apical or CSF facing membrane domain. Both processes are likely important for our understanding of the neuroinvasiveness of JCPyV.

## Materials and methods

### Cells and media

SVG-A cells (SV40 T antigen transformed glial cells-astrocyte) are a subclone of the human glial cell line SVG (SV40 T antigen transformed glial cells) transformed with an origin-defective SV40 mutant. SVG-A cells were grown in Minimum Essential Medium (Mediatech) supplemented with 10% fetal bovine serum (FBS, Atlanta Biologic) and 1% antibiotic/antimycotic (Mediatech). SVG-A cells were used for initial infections, propagation of JCPyV and generation of EV^JC+^. Primary human astrocytes (NHA) were obtained from ScienCell Research Labs and cultured in cell line-specific complete media, as indicated by the manufacturer, in a humidified incubator at 37°C with 5% CO_2_. HIBCPP cells were a gift from the Schwerk lab and were cultured as previously described [54], with minor modifications. Cells were maintained at <50% confluence in DMEM/F12 supplemented with 15% FBS, 1% anti-anti and 5ng/ml human insulin (Sigma). Cultures were kept in a humidified incubator at 37°C with 5% CO_2_. EV-depleted medium (EV-D) was used as needed for vesicle related experiments and production of infectious extracellular vesicles, while complete medium was used for culture maintenance. EV-D medium was prepared and spun at 100,000×*g* in a type 45 Ti rotor (*k* factor = 133) for 18 hours, followed by filtration through a 0.22 μm pore filter (Celltreat) [55].

### Antibodies and drugs

For indirect immunofluorescent staining of infected cells, VP1 was detected using the primary antibody PAB597 (lab-grown) followed by secondary detection with goat anti mouse Alexa-fluor 488 (ThermoFisher Scientific) and DAPI for total cell count (ThermoFisher Scientific). For the inhibitor experiments, capsaicin, chlorpromazine, dynasore, methyl-beta-cyclodextran, nystatin, EIPA and cyclosporin A were purchased from MilliporeSigma. All drugs were reconstituted in DMSO (MilliporeSigma) and stored in at -20°C. For barrier integrity experiments, sodium fluorescein and rhodamine 123 were purchased from MilliporeSigma and reconstituted in DMSO. Rhodamine was used at 10μM; cyclosporin A was used at 10μM.

### Virus and extracellular vesicles

The Mad1/SVEΔ strain of JCPyV was propagated in SVG-A cells and purified over a cesium chloride gradient as previously described [56–58]. To generate EV^JC+^, EV were concentrated by differential centrifugation of supernatant from infected SVG-A cells, as previously described [29]. SVG-A were infected with purified JCPyV and maintained in in EV-D media for seven days. Cells were trypsinized and counted on day 7 to determine viability of the culture. The supernatant containing EV^JC+^ was spun at 300×*g* in a Sorvall Legend X1R centrifuge (ThermoFisher Scientific) for 10 minutes, followed by a 2,000 × *g* spin for 10 minutes, and two 30-minute spins at 10,000 × *g* in a Sorvall Lynx 6000 centrifuge. Supernatant was then transferred to Ultra Clear tubes (Beckman Coulter) and spun at 100,000 × *g* for 2 hours, 9 minutes in a SW41 Ti rotor (*k*-factor = 124). The pellet was washed with phosphate-buffered saline (PBS) and centrifuged for an additional 2 hours at 100,000 × *g*. The pellet was suspended in sterile PBS-HAT at 1/100^th^ of the original volume. EV for short term use (less than 5 days) were stored at 4°C or for long term storage (greater than 5 days), at -80°C. All centrifugation steps were carried out at 4°C. Supernatant was transferred to a clean tube after each centrifugation step. EV preparations were characterized by western blotting and nanoparticle tracking analysis using a ZetaView Quatt (Particle Metrix) (S1 Fig). To confirm that EV^JC+^ were infectious, EV^JC+^ were used to infect SVG-A cells and VP1 was quantified at day 3 (S1 Fig). In order to quantify and normalize input JCPyV and EV^JC+^ for barrier pass through experiments, the concentration of protected genome copies/mL of virus and EV^JC+^ preparations was determined by quantitative PCR using a VP2 primer-probe set and comparison to a standard curve. Specific conditions are detailed in the quantitative PCR section.

### Western blotting

For western blotting, HSP70 and GM130 (Cell Signaling Technology) were used to detect vesicles and contaminating cellular protein, respectively (S1 Fig). Primary antibodies were diluted 1:1,000 (HSP70 and GM130) or 1:2,500 (PAB597) in 1X Tris buffered saline with 2% BSA and incubated overnight at 4°C, with rocking. Following primary antibody incubation, blots were washed for 5 minutes three times in 1X TBS with 0.01% Tween (TBS-T). Primary antibodies were detected with HRP-linked goat anti-mouse and goat anti-rabbit secondary antibodies (ThermoFisher) diluted 1:10,000 in 1X TBS for one hour at room temperature, in the dark. Blots were washed in TBS-T an additional three times for 5 minutes each. Secondary antibodies were detected using ClarityMax chemiluminescent detection reagent (BioRad) and visualized using a ChemiDoc Detection System (BioRad).

### Naïve barrier culture

HIBCPP cultures between passages 30–38 were used for experiments. Prior to subculture, 6.5mm polyethylene (PET), pore size 0.4μm, transwell inserts (Corning) were equilibrated with 600μl complete HIBCPP media in the basal chamber. Transwell inserts with media were warmed in a 37°C, 5% CO_2_ incubator until ready to use. HIBCPP cells were subcultured using 1ml 0.25% trypsin (Corning) per 25 cm^2^ flask surface area at 37°C for 15 minutes. Cells were resuspended to a final concentration of 2e^6^ cells/ml in complete media (FBS concentration = 15%). Transwell inserts were seeded with 750,000 cells/cm^2.^ 24 hours after seeding, media in the apical and basal chambers was replaced with DMEM/F12 containing 10% FBS, 1% A/A and 5ng/ml insulin. Transepithelial electrical resistance (TEER) was monitored daily beginning 48 hours after seeding. At 72 hours, media in the apical and basal chambers was replaced with DMEM/F12 containing 5% FBS, 1% A/A and 5ng/ml insulin and monitored by checking TEER daily until >300 Ω*cm^2^ was achieved.

### Infected barrier culture

HIBCPP cultures between passages 30–38 were used for experiments. Cells were seeded at a density of 250,000 cells/cm^2^ in 6-well tissue culture treated plates. Cells were infected with purified JCPyV (MOI = 100) for 2 hours at 37°C or mock infected with virus free, unsupplemented media. Following infection, virus containing media was removed and replaced with DMEM/F12 containing 5% FBS, 1% A/A and 5ng/ml insulin. At three days post infection, infected and mock infected cells were subcultured using 1ml 0.25% trypsin and resuspended to a final concentration of 2e^6^ cells/ml in complete media (FBS concentration = 5%). Cultures were plated to 96-well tissue culture plates and transwell inserts were seeded with 750,000 cells/cm^2.^ TEER was measured daily starting at 4 days post infection. 20uL samples of media from the apical and basal chambers were collected daily, prior to TEER readings. Supernatant from 7 days post infection was incubated with naïve SVG-A cultures and allowed to spread over several rounds of replication.

### Transepithelial electrical resistance measurements

Transepithelial electrical resistance (TEER) was measured in every experimental well using an EVOM-2 Voltohmmeter by placing one probe in the basal chamber and one probe in the apical chamber until the reading stabilized. Transwell plates were allowed to equilibrate at room temperature for 20 minutes prior to reading. Electrodes were equilibrated in room temperature 1X PBS for 5 minutes prior to reading sample TEER. The electrode was sterilized by submerging in ethanol between wells. TEER was measured daily prior to samples collections and media changes. Ω*cm^2^ was calculated by subtracting the measured value of transwell inserts without cells in media (“cell free”) from the measured value of the sample wells, and multiplying by the transwell surface area.

### Sodium fluorescence penetrance assay

HIBCPP cells were cultured 24 well, 0.4μm PET transwell inserts for 4 days as described above, until a barrier was formed. TEER for time zero was measured and media was replaced with unsupplemented media (DMEM/F12) in the basal chamber. 10μM sodium fluorescein in DMEM/F12 was added to the apical chamber. At 15, 30, 45 and 60 minutes post addition, media from the basal chamber was collected and replaced with DMEM/F12, to maintain the starting volume. After 60 minutes, an additional sample was collected from the apical chamber. Sodium fluorescein accumulation was measured using a BioTek Cytation 5 plate reader. DMEM/F12 alone was measured to determine background.

### Directional transport assay

HIBCPP cells were cultured 24 well, 0.4μm PET transwell inserts for 4 days as described above, until a barrier was formed. TEER for time zero was measured and media was replaced with basal media (DMEM/F12) containing 10μM cyclosporin A (CsA) or vehicle control (DMSO, 0.0001μl/ml) for one hour at 37°C. Following pretreatment, 10μM rhodamine 123 was added in the presence of CsA or vehicle. Transport of rhodamine was measured in both the apical➔basal and basal➔apical direction, at one and three hours post addition of rhodamine, and permeability coefficients were calculated as previously described [59, 60]. Rhodamine accumulation was measured using a BioTek Cytation 5 plate reader for accumulated fluorescence.

### Infection assays

SVG-A cells or NHA cells were plated at 5,000 cells/cm^2^. Supernatant containing JCPyV or EV^JC+^ from the apical and basal chambers were used to infect naïve cultures for 2 hours. Following infection, virus containing media was removed and replaced with complete media or complete EV-D media as appropriate per cell type. Cells were incubated 37°C and stained for VP1 at 3 days (SVG-A) 5–15 days (NHA) post infection.

### Toxicity

HIBCPP cells were plated at 10,000 cells/cm^2^ in 96 well plates (Corning) in phenol free DMEM/F12. Drugs were resuspended in DMSO and incubated with cells at the following concentrations: EIPA 100μM; chlorpromazine 100μM; dynasore 100μM; methyl-beta-cyclodextran 5mM; nystatin 5μM; capsaicin 30μM and 60μM. Volume-matched DMSO was used as a vehicle control. At 2 hours, 24 hours, and 48 hours post addition, viability was assayed using a Viral ToxGlo kit (Promega), according to the manufacturer’s protocol. Luciferase activity was measured with a GloMax Multidetection plate reader (Promega).

### Indirect immunofluorescence

To quantify infected cells, cultures were fixed in 100% ice cold methanol (MeOH), and incubated at −20°C for 30 minutes. Fixed cells were rehydrated in 1x PBS for 15 minutes at room temperature, and incubated with a VP1-specific antibody PAB597 (1:50) in PBS at 37°C for 1 hour. Following incubation cells were washed twice with PBS and incubated with a cocktail of DAPI (ThermoFisher, 1:1,000 dilution) and goat anti-mouse Alexa fluor 488 (ThermoFisher, 1:1,000 dilution) secondary antibody in PBS at 37°C for an additional hour. Cells were analyzed for nuclear VP1 and total cell number under a 20x objective using a Ti2-E fluorescent microscope (Nikon). Total and infected cell counts were quantified with Elements High Content Imaging software (Nikon).

### Quantitative PCR

Protected viral genome content of apical and basal supernatants was measured by quantitative PCR as previously described [29]. Supernatants were first treated with DNAse 1 (New England Biolabs) for 30 minutes at 37°C, followed by a 10 minute inactivation at 75°. DNA was extracted using a DNeasy96 Blood and Tissue Kit (Qiagen). qPCR was conducted using a VP2 Taqman Assay primer/probe set (probe: /5HEX/TGTTCTCCA/ZEN/CAATCTCCCAGGCTT/3IABkFQ/ primer 1: CCTGGAGTGAATGCCTTTGT primer 2: AGAGGTTAAGGCTGGCAAATC) (IDT) and run on a CFX96 Detection system (BioRad). Genome number was calculated by comparison to a standard curve of JCPyV DNA.

### Barrier infiltration

For viral and EV pass through experiments using transcytosis inhibitors, the basolateral side of HIBCPP cultures were pretreated with the indicated inhibitor for 2 hours at 37°C in unsupplemented DMEM/F12 media. Following pretreatment, equivalent GE/ml of JCPyV or EV^JC+^ were added to the basolateral chamber in the continued presence of drug. In the cyclosporin A (CsA) treated transwells, cultures were pretreated with CsA for 1 hour at 37°C. Following CsA, EIPA 100μM; chlorpromazine 100μM; dynasore 100μM; methyl-beta-cyclodextran 5mM; nystatin 5μM was added to the basolateral chamber in the continued presence of CsA for an additional 2 hours. Supernatant from the apical and basal chambers of JCPyV or EV^JC+^ treated, JCPyV or EV^JC+^ plus transcytosis inhibitors, and JCPyV or EV^JC+^ plus inhibitors and CsA were collected at 24 or 48 hours post addition. The TEER of all wells was measured at time zero and prior to collecting supernatant.

### Statistics and calculations

Cell count analysis was performed using Elements High Content imaging software (Nikon). A Student’s *t*-test was used to calculate the p-value of each experiment using Microsoft Excel. A *p*-value of less than 0.05 was considered significant and is indicated in figures by *. Where there is no significant change, *p* > 0.05, the figure legend indicates NS for no significance. Infection is reported on the median value of VP1+ cells. Error bars represent the standard deviation from 3 independent experiments. Underlying data for all figures are provided as supplementary data (S1 Data).

To calculate sodium fluorescein penetrance and directional transport, background was subtracted from all fluorescence readings prior to calculation. Sodium fluorescein penetrance was calculated as previously described [61, 62]:

$$SR,T=(RFU30min+(RFU15min*\frac{75\mu l}{750\mu l}))$$

$$Clearance\;volume=\frac{\left(Vb*SB,T\right)}{St,60\;min}$$

$$PE\left(\frac{cm}{min}\right)=\left[(\frac{1}{1PE})\right)/1000]/Area$$

Efflux was calculated using the following equation, as previously described [59]:

$$Papp,A\to B=\frac{RFU\;basal\;chamber}{RFU\;apical\;chamber*area*time}$$

$$Papp,B\to A=\frac{RFU\;apical\;chamber}{RFU\;basal\;chamber*area*time}$$

$$Efflux\;Ratio=\frac{Papp,B\to A}{Papp,A\to B}$$

## Supporting information

S1 Fig Characterization of EV^JC+^. A) EV from infected SVG-A cultures were concentrated by differential centrifugation at seven days post infection (DPI), and resuspended in 1X PBS-HAT using 1/100^th^ of the collected media volume. B) Particle count and size were measured using nanoparticle tracking analysis on a ZetaView Quatt. C) EV from infected cultures are positive for the EV marker HSP70, positive for viral protein 1 (VP1), and negative for the cellular contamination marker GM130. D) EV^JC+^ and naïve EV were used to infect SVG-A cells. EV are infectious as shown by indirect immunofluorescent staining for VP1 at 3 days post infection. DAPI (total cells) is shown in blue and VP1 (infected cells) is shown in green.
(TIF)

S2 Fig Relative toxicity of capsaicin and impact on JCPyV infection. A) Relative toxicity of capsaicin. HIBCPP cells were cultured to confluence and treated @ 37°C with 30 or 60μM capsaicin in unsupplemented DMEM/F12 media. After 24h, cell viability was quantified using a luciferase-based viability kit (ToxGlo, Promega), according to the manufacturer’s protocol. B) Capsaicin does not inhibit infection. SVG-A cells were pretreated with vehicle (DMSO), 30μM or 60μM capsaicin for 2h, following by infection with purified JCPyV. Pretreatment with capsaicin did not interfere with infection under the tested conditions. UI = uninfected control; NS = not significant.
(TIF)

S3 Fig Toxicity and efficacy of transcytosis inhibitors. A) HIBCPP cells were cultured to confluence in 96-well dishes and treated @ 37°C with transcytosis inhibitors in phenol-free media. At 2, 24, and 48 hours post addition, cell viability was quantified using a luciferase-based viability kit (ToxGlo, Promega). Individual dosing is as follows: EIPA 100μM; Chlorpromazine 100μM; Dynasore 100μM; MBCD 5mM; Nystatin 5μM. DMSO was used as a vehicle control, volume matched to the highest concentration present. All compounds were non-toxic at the doses used. B) HIBCPP cells were cultured to confluence in 24-well dishes and treated @ 37°C with transcytosis inhibitors for two hours, followed by incubation with fluorescent dye controls in the presence of drug for an additional two hours. Transferrin-633 was used as a control for uptake by clathrin dependent endocytosis; Cholera toxin subunit B-488 was used as a control for uptake by raft mediated endocytosis; Dextran-488 was used as a control for uptake by macropinocytosis. Following incubations, cells were collected using trypsin, washed extensively, and analyzed by flow cytometry for intracellular fluorescence. Chlorpromazine, dynasore, MßCD and EIPA significantly reduced the uptake of control molecules. * = p < 0.05, NS = not significant.
(TIF)

S4 Fig Barrier integrity controls for Fig 5; the effect of transcytosis inhibitors on TEER and sodium fluorescein penetrance in the presence of purified JCPyV. HIBCPP cells were cultured on transwell inserts as described. Unsupplemented DMEM/F12 media containing transcytosis inhibitors or vehicle alone was added to transwells, in triplicate. Time zero and +24 hour TEER values (left axis, panels A-E, black and gray bars) were measured for all samples. At +24 hours post addition, a sodium fluorescein assay was used to determine the impact that inhibitors may have had on penetrance (PE, right axis, panels A-E, gray line). A) Penetrance and TEER following 100μM chlorpromazine exposure. B) Penetrance and TEER following 5mM methyl-beta-cyclodextran exposure. C) Penetrance and TEER following 100μM EIPA exposure. D) Penetrance and TEER following 100μM dynasore exposure. E) Penetrance and TEER following 5μM nystatin exposure.
(TIF)

S5 Fig Barrier integrity controls for Fig 6; the effect of transcytosis inhibitors on TEER and sodium fluorescein penetrance in the presence of EV^JC+^. HIBCPP cells were cultured on transwell inserts as described. Unsupplemented DMEM/F12 media containing transcytosis inhibitors or vehicle alone was added to transwells, in triplicate. Time zero and +24 hour TEER values (left axis, panels A-E, black and gray bars) were measured for all samples. At +24 hours post addition, a sodium fluorescein assay was used to determine the impact that inhibitors may have had on penetrance (PE, right axis, panels A-E, gray line). A) Penetrance and TEER following 100μM chlorpromazine exposure. B) Penetrance and TEER following 5mM methyl-beta-cyclodextran exposure. C) Penetrance and TEER following 100μM EIPA exposure. D) Penetrance and TEER following 100μM dynasore exposure. E) Penetrance and TEER following 5μM nystatin exposure.
(TIF)

S6 Fig JCPyV is detectable by qPCR following incubation with physiologically relevant concentrations of virus. HIBCPP cells were cultured on transwell inserts as described. Viral genome concentration of the virus stock was quantified by qPCR. JCPyV was added to the basolateral chamber of HIBCPP barriers and cell free controls, in a dose curve starting at 10^4^ protected genome equivalents/ml. 24h later, supernatant from the apical chamber was collected and analyzed by qPCR for protected genome content/ml.
(TIF)

S1 Data Underlying data for Figs 1–7 and S1–S6 Figs. (XLSX)
