# Supplementary figures and images for: Highly restrictive and directional penetration of the blood cerebral spinal fluid barrier by JCPyV
Source: PLoS Pathog. 2024 Jul 22;20(7):e1012335. doi: 10.1371/journal.ppat.1012335 (PMC11293668; doi:10.1371/journal.ppat.1012335)

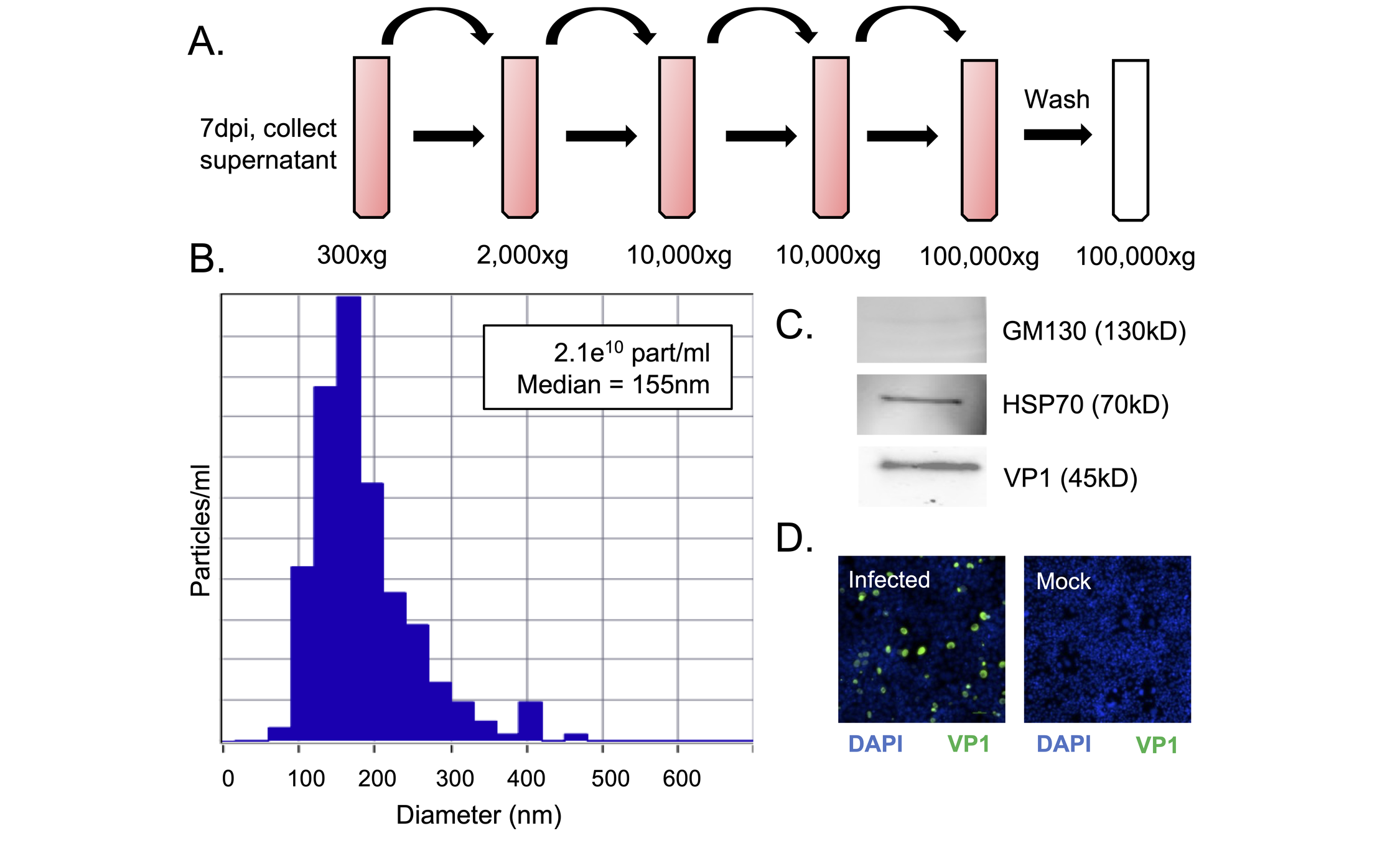

Supplement: S1 Fig — A) EV from infected SVG-A cultures were concentrated by differential centrifugation at seven days post infection (DPI), and resuspended in 1X PBS-HAT using 1/100th of the collected media volume. B) Particle count and size were measured using nanoparticle tracking analysis on a ZetaView Quatt. C) EV from infected cultures are positive for the EV marker HSP70, positive for viral protein 1 (VP1), and negative for the cellular contamination marker GM130. D) EVJC+ and naïve EV were used to infect SVG-A cells. EV are infectious as shown by indirect immunofluorescent staining for VP1 at 3 days post infection. DAPI (total cells) is shown in blue and VP1 (infected cells) is shown in green. (TIF) [file ppat.1012335.s001.tif]

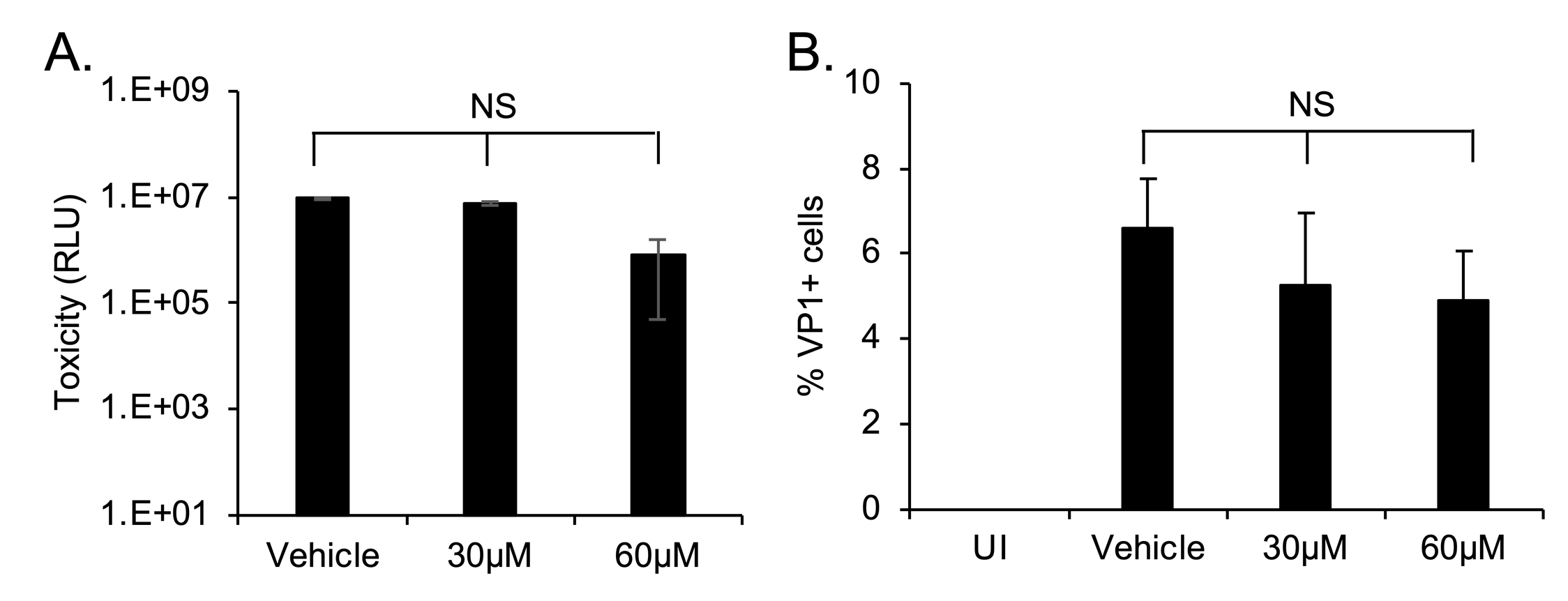

Supplement: S2 Fig — A) Relative toxicity of capsaicin. HIBCPP cells were cultured to confluence and treated @ 37°C with 30 or 60μM capsaicin in unsupplemented DMEM/F12 media. After 24h, cell viability was quantified using a luciferase-based viability kit (ToxGlo, Promega), according to the manufacturer’s protocol. B) Capsaicin does not inhibit infection. SVG-A cells were pretreated with vehicle (DMSO), 30μM or 60μM capsaicin for 2h, following by infection with purified JCPyV. Pretreatment with capsaicin did not interfere with infection under the tested conditions. UI = uninfected control; NS = not significant. (TIF) [file ppat.1012335.s002.tif]

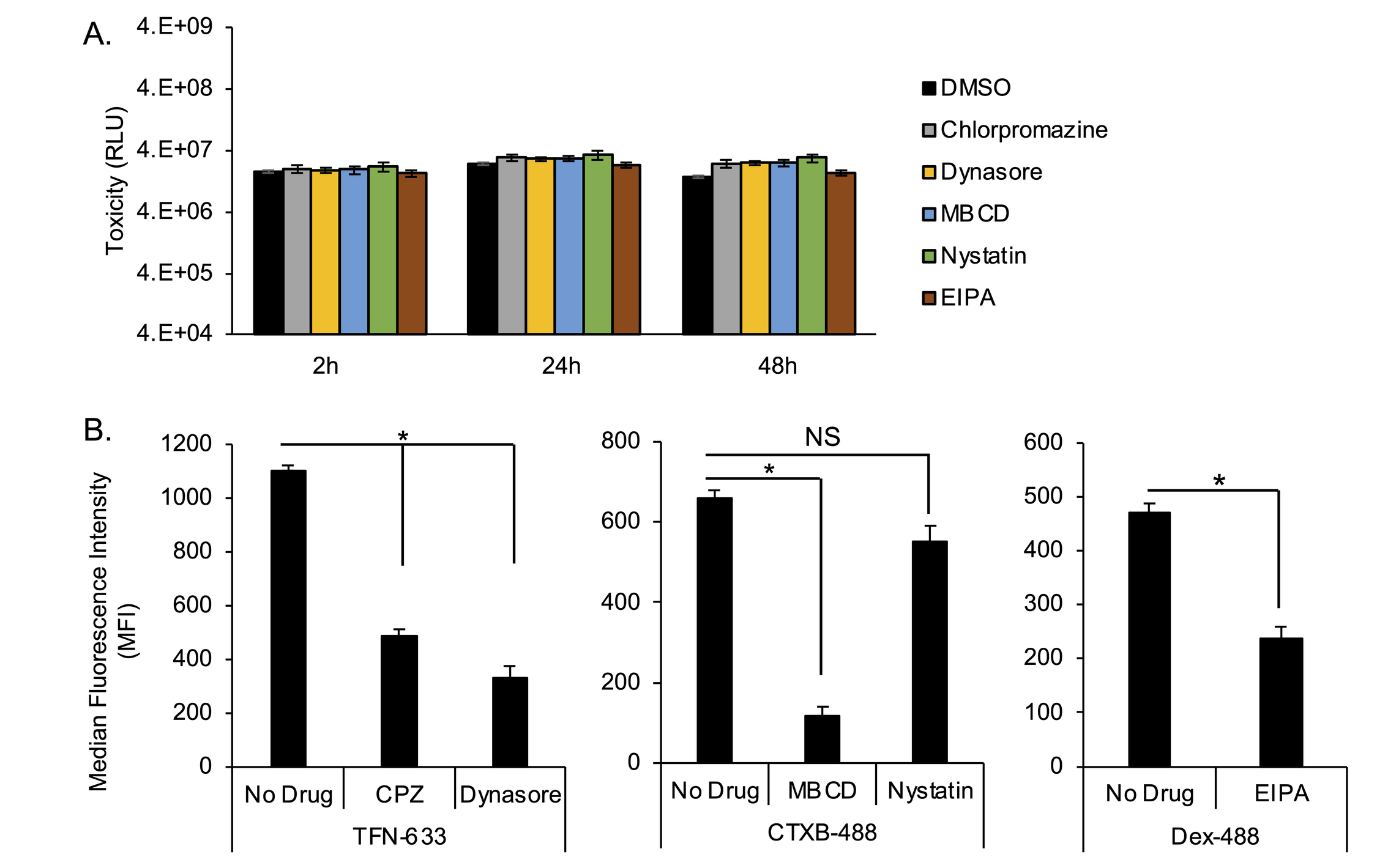

Supplement: S3 Fig — A) HIBCPP cells were cultured to confluence in 96-well dishes and treated @ 37°C with transcytosis inhibitors in phenol-free media. At 2, 24, and 48 hours post addition, cell viability was quantified using a luciferase-based viability kit (ToxGlo, Promega). Individual dosing is as follows: EIPA 100μM; Chlorpromazine 100μM; Dynasore 100μM; MBCD 5mM; Nystatin 5μM. DMSO was used as a vehicle control, volume matched to the highest concentration present. All compounds were non-toxic at the doses used. B) HIBCPP cells were cultured to confluence in 24-well dishes and treated @ 37°C with transcytosis inhibitors for two hours, followed by incubation with fluorescent dye controls in the presence of drug for an additional two hours. Transferrin-633 was used as a control for uptake by clathrin dependent endocytosis; Cholera toxin subunit B-488 was used as a control for uptake by raft mediated endocytosis; Dextran-488 was used as a control for uptake by macropinocytosis. Following incubations, cells were collected using trypsin, washed extensively, and analyzed by flow cytometry for intracellular fluorescence. Chlorpromazine, dynasore, MßCD and EIPA significantly reduced the uptake of control molecules. * = p < 0.05, NS = not significant. (TIF) [file ppat.1012335.s003.tif]

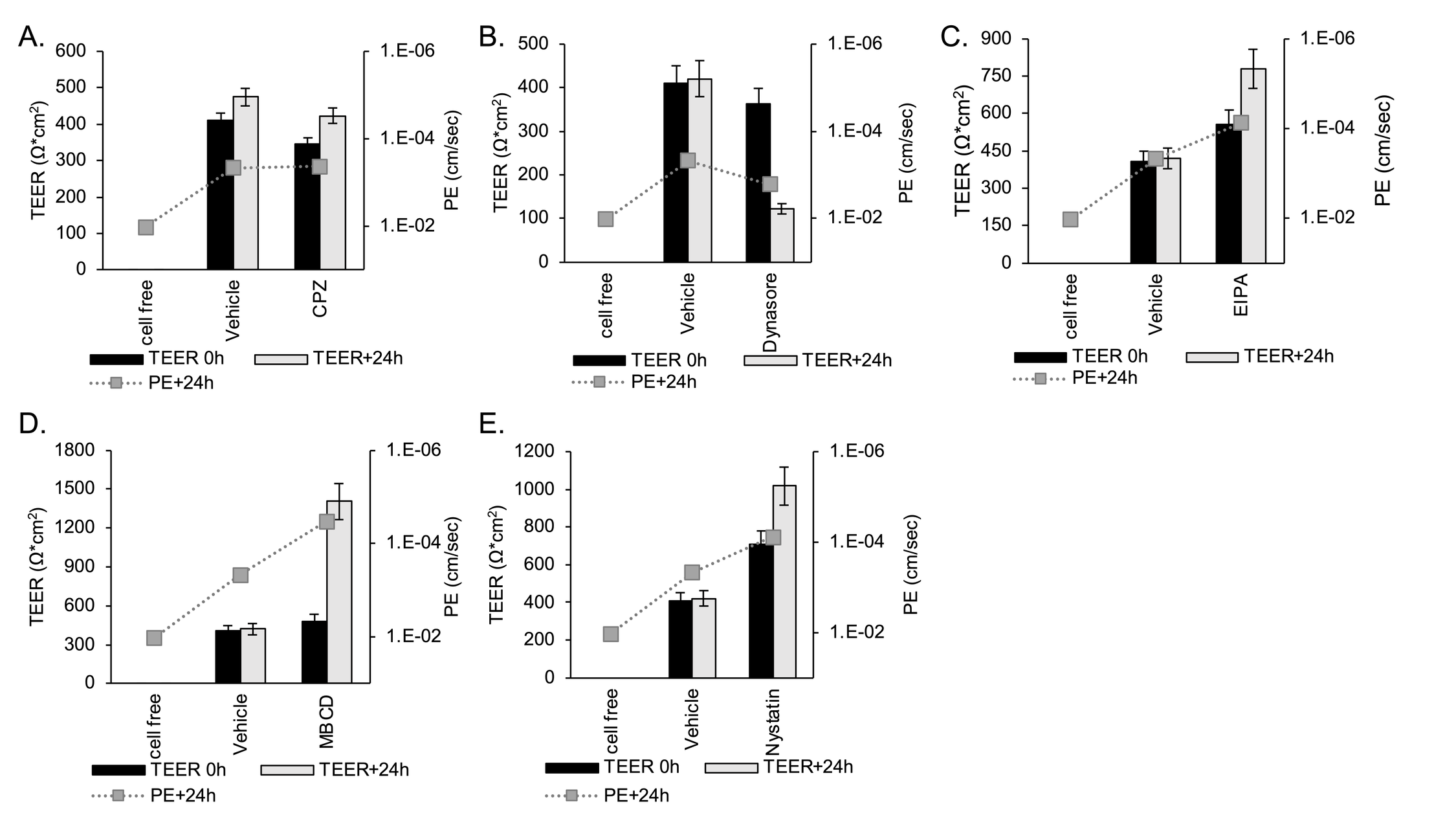

Supplement: S4 Fig — HIBCPP cells were cultured on transwell inserts as described. Unsupplemented DMEM/F12 media containing transcytosis inhibitors or vehicle alone was added to transwells, in triplicate. Time zero and +24 hour TEER values (left axis, panels A-E, black and gray bars) were measured for all samples. At +24 hours post addition, a sodium fluorescein assay was used to determine the impact that inhibitors may have had on penetrance (PE, right axis, panels A-E, gray line). A) Penetrance and TEER following 100μM chlorpromazine exposure. B) Penetrance and TEER following 5mM methyl-beta-cyclodextran exposure. C) Penetrance and TEER following 100μM EIPA exposure. D) Penetrance and TEER following 100μM dynasore exposure. E) Penetrance and TEER following 5μM nystatin exposure. (TIF) [file ppat.1012335.s004.tif]

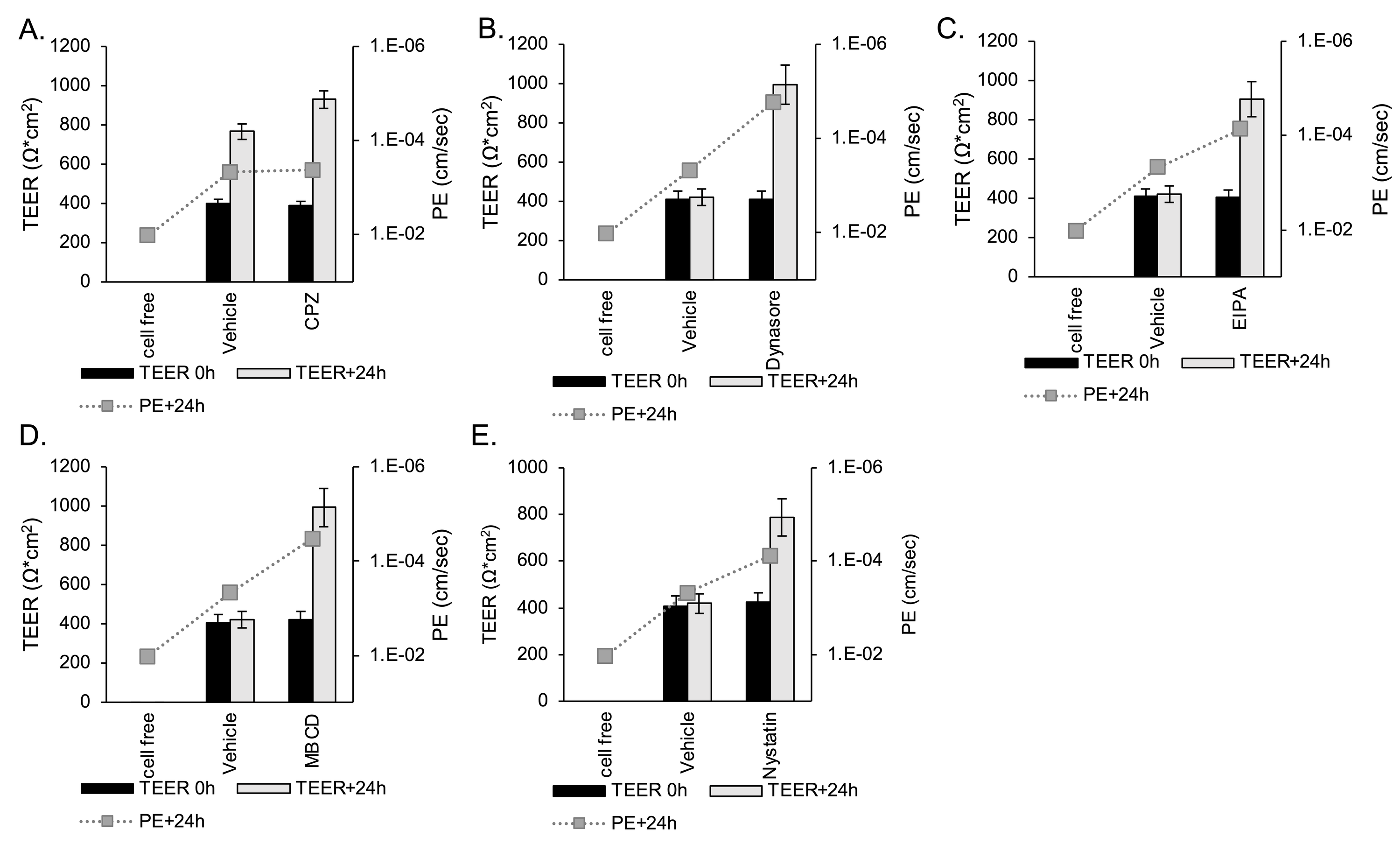

Supplement: S5 Fig — HIBCPP cells were cultured on transwell inserts as described. Unsupplemented DMEM/F12 media containing transcytosis inhibitors or vehicle alone was added to transwells, in triplicate. Time zero and +24 hour TEER values (left axis, panels A-E, black and gray bars) were measured for all samples. At +24 hours post addition, a sodium fluorescein assay was used to determine the impact that inhibitors may have had on penetrance (PE, right axis, panels A-E, gray line). A) Penetrance and TEER following 100μM chlorpromazine exposure. B) Penetrance and TEER following 5mM methyl-beta-cyclodextran exposure. C) Penetrance and TEER following 100μM EIPA exposure. D) Penetrance and TEER following 100μM dynasore exposure. E) Penetrance and TEER following 5μM nystatin exposure. (TIF) [file ppat.1012335.s005.tif]

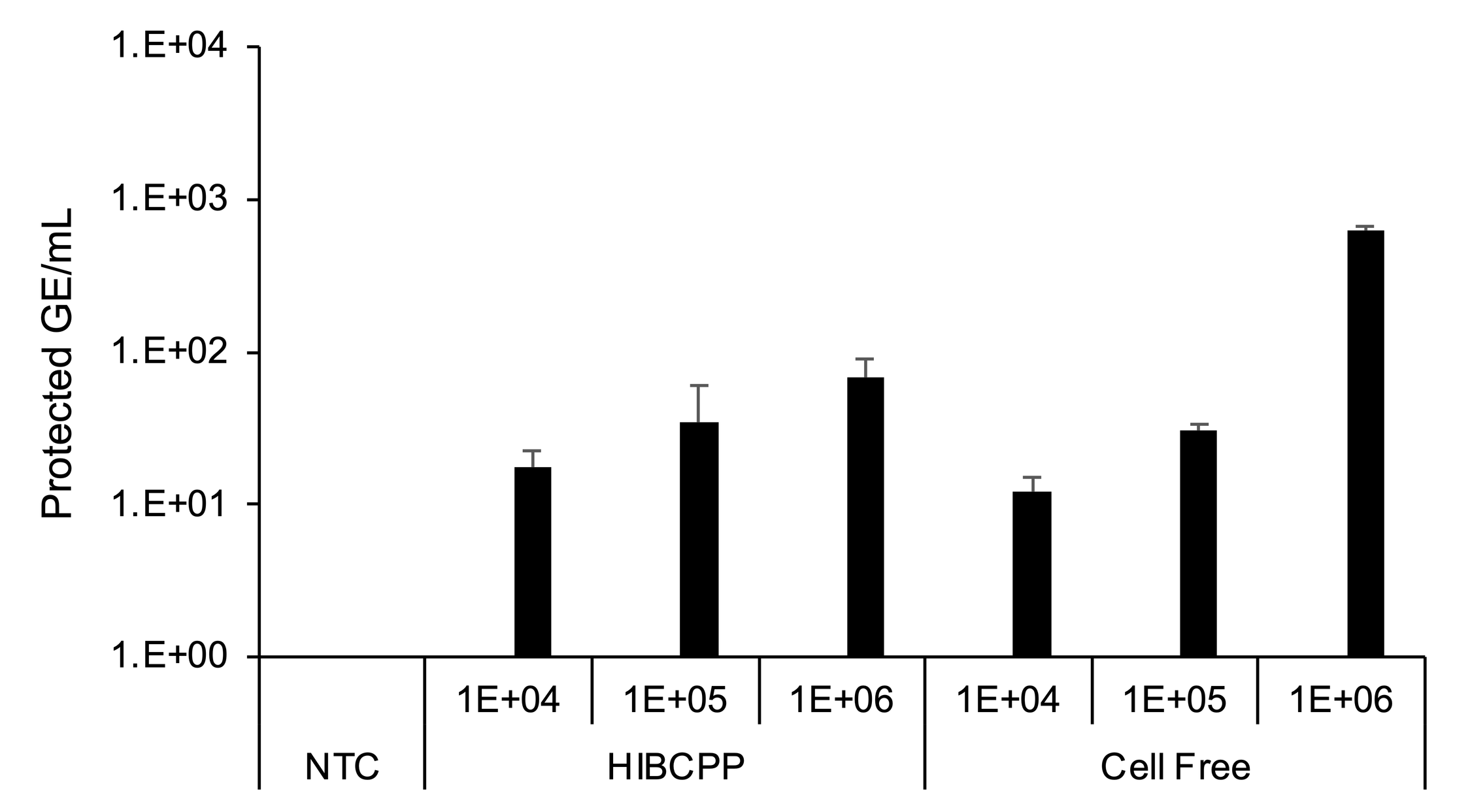

Supplement: S6 Fig — HIBCPP cells were cultured on transwell inserts as described. Viral genome concentration of the virus stock was quantified by qPCR. JCPyV was added to the basolateral chamber of HIBCPP barriers and cell free controls, in a dose curve starting at 104 protected genome equivalents/ml. 24h later, supernatant from the apical chamber was collected and analyzed by qPCR for protected genome content/ml. (TIF) [file ppat.1012335.s006.tif]
